# Can Bacteriophages Replace Antibiotics?

**DOI:** 10.3390/antibiotics11050575

**Published:** 2022-04-26

**Authors:** Mikael Skurnik

**Affiliations:** 1 Department of Bacteriology and Immunology, Medicum, Human Microbiome Research Program, Faculty of Medicine, University of Helsinki, 00290 Helsinki, Finland; mikael.skurnik@helsinki.fi; Tel.: +358-50-3360981; 2 Division of Clinical Microbiology, HUSLAB, University of Helsinki and Helsinki University Hospital, 00290 Helsinki, Finland

**Keywords:** antibiotic resistance, bacteriophage, phage therapy

## Abstract

Increasing antibiotic resistance numbers force both scientists and politicians to tackle the problem, and preferably without any delay. The application of bacteriophages as precision therapy to treat bacterial infections, phage therapy, has received increasing attention during the last two decades. While it looks like phage therapy is here to stay, there is still a lot to do. Medicine regulatory authorities are working to deliver clear instructions to carry out phage therapy. Physicians need to get more practical experience on treatments with phages. In this opinion article I try to place phage therapy in the context of the health care system and state that the use phages for precision treatments will require a seamless chain of events from the patient to the phage therapy laboratory to allow for the immediate application of phages therapeutically. It is not likely that phages will replace antibiotics, however, they will be valuable in the treatment of infections caused by multidrug resistant bacteria. Antibiotics will nevertheless remain the main treatment for a majority of infections.

## 1. Introduction

Bubonic plague, pertussis, diphtheria, pulmonary consumption or tuberculosis, typhoid and cholera are still ominous names of lethal infectious diseases caused by diverse bacterial pathogens. Prior to the invention of antibiotics, bacterial infections killed humans irrespective of age or sex [1]. Often the causative agent of the infection gained access to the bodily organs through wounds, insect bites or in contaminated or spoiled foodstuffs. The most dangerous contagious diseases spread between humans due to bad hygiene, contact transmission or via aerosols released by coughing and sneezing, and could cause extremely wide epidemics and pandemics, with one of the deadliest being the medieval Black Death in Europe, caused by the plague bacillus, *Yersinia pestis* [2,3,4]. Since the beginning of the 20th century, vaccinations along with an improved general level of hygiene significantly reduced the number of people succumbing to bacterial infections [5]. This trend continued strongly, and was supported by the invention and use of antibiotics during World War II [6]. The availability and use of antibiotics was experienced as the final victory over the infectious diseases caused by bacteria [7].

Already in the 1950s, more and more bacteria resistant to the earliest used antibiotics were isolated from patient samples. The inventor of penicillin and Nobel prize laureate Sir Alexander Fleming warned in his Nobel-seminar in 1945 that bacteria will develop resistance to antibiotics, especially if the treatment regime is undersized [8]. Thus, very soon after the use of novel antibiotics for therapeutics, with a short lag, the number of bacterial isolates resistant to those drugs has started to increase [9]. In fifty years, on the shift of the millennium, it has become evident that the winners of this race will be the bacteria. The development of new antibiotics is slow and expensive, and their relatively short time window of use due to the inevitable appearance of resistance does not make their development as the first priority for big pharma.

## 2. Are There Alternatives to Antibiotics?

Humans live in a world where there are bacteria everywhere, and it is impossible to avoid encountering them. Most of the bacteria cause no harm to humans, while some of the bacteria are symbiotic and thereby even beneficial and necessary for the wellbeing of them. On the other hand, some bacteria have specialized to use the human body as their source of vital nutrients or growth media. This has become possible for the bacteria during the evolution through the acquisition of special virulence or pathogenicity factors that enable the specialized pathogens to defend against or evade the immune defense mechanisms of the human body, and thereby the pathogens can thus colonize the humans [10]. Different bacteria have specialized to different tissues and organs of the human body. The bacteria generally have adhesion factors that allow them to bind to tissues and cells, thereby preventing their elimination from the tissues. Bacteria need invasion factors to penetrate into tissues either through cells or between the cells. When within the tissues, in the blood stream, or in extracellular fluid, bacteria need specific virulence factors to defend against the complement system and the phagocytes. Based on the set-up of the virulence factors, different bacteria are specialized to infect different organs and tissues. Thereby, some bacteria cause intestinal infections, while others cause sepsis, pneumonia or sexually transmitted diseases.

The pathogens also differ based on their infective doses. For some bacteria it is very low, even one single bacterium may be enough to start the infection; however, for other bacteria the dose may be high, even millions of bacteria [11]. For example, the causative agents of bubonic plague and dysentery, *Yersinia pestis* and *Shigellae*, respectively, are contagious in very small numbers. 

Since the causative agents of bacterial infections are highly diverse, their prevention and treatment also requires diverse actions. The spreading of some bacterial infections can be prevented by improved hygiene and better living standards, therefore, a functional waste management system and the availability of clean water plays a central role. The improvement of these two factors may specifically decrease the number of intestinal infections. In addition, malnourishment of children poses an increased risk to infections. 

Vaccination is an efficient measure to prevent bacterial infections caused by pathogens that demonstrate little antigenic variability between the bacterial isolates. Such pathogens are, for example, those included in the current vaccination programs. The vaccine containing the weakened toxins produced by *Corynebacterium diphtheria* or *Clostridium tetani* provide protection against diphtheria or tetanus, respectively, or the virulence factors of *Bordetella pertussis* protect against pertussis.

The vaccination program also includes a vaccine against pneumococci that is a result of a compromise, as there are over 100 known capsule types among pneumococcus strains. Among those the 23 most common capsule types that cause human infections have been chosen [12]. To prevent infections caused by these types, a combination vaccine containing those antigens was developed. The vaccination programs also include a vaccine against *Haemophilus influenza* bacteria that causes respiratory and other severe infections. There are also vaccines against the causative agents of tuberculosis, bubonic plague, cholera, typhoid, tularemia and anthrax, to mention but a few. On the other hand, the production of vaccines against many other pathogens has not been successful. This may be due to the nature of the infection or the fact that the bacterial species in question has a vast number, even hundreds, of antigenic variants, making the production of vaccines not feasible.

## 3. Are There Any Other Ways to Co-Exist with Bacteria? 

Prebiotics and synbiotics have been proposed as a possible solution to manage intestinal infections [13]. Probiotics could be referred to as the “good” or “beneficial” bacteria, the good guys. The normal microbiota is important for the wellbeing of the body, as it can to some extent prevent the colonization efforts of their ecological niches like the gut or the mucosal surfaces by the invading pathogens. It is believed that increasing the number of the good bacteria via probiotics could better maintain the correct balance of the body microbiota and thereby prevent bacterial infections. Faecal microbial transplantations (FMTs) can be regarded as one variant of probiotics [14]. In FMT a suspension of the stools from a healthy donor is introduced into the gut of the patient that has been super-infected, for example, after antibiotic treatment, by *Clostridioides difficile*, and can cause very severe symptoms. FMT may revert the normal balance of the gut microbiome and eradicate *C. difficile*. It has been reported that FMT may also be a viable alternative to treat disturbations of the gut caused by other bacteria. Analogously, vaginal infections have been addressed by vaginal microbiota transplantations [15]. An interesting observation concerning FMTs has been that the removal of bacteria and bigger microbes from the transplant material by filtration has not decreased the efficacy of the treatment [16]. This implies that bacteriophages present in the transplant play a big role in maintaining the balance in the gut.

## 4. What Are Bacteriophages and Might They Be a Noteworthy Alternative to Antibiotics?

Bacteriophages (phages) are viruses that are specialized to infect bacteria. It has been estimated that there is an unbelievable number (10^31^) of phages in the world, a 10-fold excess over the estimated number of bacteria [17]. Phages are a highly diverse group that was initially classified based on their morphology detected under transmission electron microscopy. Most known phages have a structure containing a head or capsid to which the phage packages its genomic material, and a tail that the phage uses to adsorb on its host bacterium, and through which it injects the genetic material into the cytoplasm of the bacterial cell. There are also tail-less phages, and filamentous ones in which the genome is protected by a helical protein layer wrapped around the nucleic acid.

The life cycle of a phage resembles that of animal viruses. It starts by the adsorption of the phage on the bacterial surface via the recognition of the receptor structure by the phage receptor binding protein that is usually located at the distal tip of the tail fibers or tail spike proteins. Tail-less phages carry the receptor binding structure directly on the capsid. After the phage has firmly attached on the bacterial surface, it will eject its genome through the cell wall into the bacterial cytoplasm. The genome is activated in the cytoplasm, and a series of events will follow where various gene products of the phage will be expressed. With them the phage will take control over the bacterial cell and start reproducing itself in a carefully controlled chain of events. When the new phage particles are fully completed (the phage may produce from tens to thousands of copies of itself within a single bacterial cell), they will be released from the bacterial cell by a specifically dedicated lysis system composed of lysins and holins. After receiving an exactly timed activation signal, the lysis system breaks the cell membranes and the cell wall of the bacterium, thereby releasing the phage progeny to the environment [18]. This also finally kills the bacterium. The released phage particles will then float around until they find a new host bacterium to repeat the life cycle again. This will continue as long there are host bacteria around. Phages do not move actively and are therefore under the mercy of their environmental possibilities and will encounter the new host bacteria in the surrounding liquid or soil completely by chance. One consequence of this is that the phages have during their long evolution become very sturdy and can wait for the appearance of a new host bacterium for a long time without losing their infectivity.

A continuous arms race between bacteria and phages is going on in nature [19]. The bacteria defend themselves with changes in their surface structures and/or by developing various defense mechanisms to destroy the phage-injected nucleic acid [20,21]. This phenomenon leads to the emergence of phage-resistant bacterial strains. Interestingly, from the perspective of phage therapy, the emergence of phage-resistant mutants has not been a serious problem in most patient cases. This is partly due to the fact that the loss of a phage receptor on the bacterial surface is often associated with reduced virulence as these surface structures, like LPS, capsules, and membrane proteins, are also virulence factors of the pathogens [22]. One can also consider the topic theoretically. The spontaneous point mutations occur at a rate of 10^−10^ to 10^−9^ per nucleotide per generation for bacteria [23]. Thus, at any time in the body of the patient, there may emerge a few bacterial mutants that are resistant to the phage. Provided that the phages could eradicate 99.99% of the pathogens, the remaining 0.01% plus the spontaneous phage resistant mutants would be easily taken care of by the immune system [24]. The situation may be different with immunocompromised patients.

It is possible that during phage therapy, virulent phage-resistant mutants are selected, as was the situation in the Tom Patterson case [25]. In such a case, there are several options to tackle the problem. One obvious approach is to identify phages that can infect the mutant, either from the available collection of phages, or by the selection of spontaneous host-range mutants originating from available phages [26].

In nature, phages respond to bacterial defense strategies flexibly by changes that retain their infectivity. There is a consensus that one role of the phages in nature is to keep a balance between bacterial populations to prevent uncontrolled increases in the bacterial numbers. It has been estimated that 10^23^ phage infections occur every second [27]. It has to be noted that in nature the phages never kill all their host bacteria, since the phages start to have difficulties in finding new hosts when the concentration of the target bacteria drops below 10^4^/mL or g [28,29]. This critical concentration is called the “phage proliferation threshold”. One can imagine that in nature the concentrations of bacteria and their phages follow each other as overlapping sine waves. The increase in bacterial concentrations is followed with some delay by the increase in phage concentrations that subsequently will cause the decrease in bacterial concentrations again followed by decreases in the phage concentrations [30]. This way neither organism is completely eradicated and the cycles will continue keeping the numbers of both bacteria and phages in balance. With respect to phage therapy, the proliferation threshold implies that phages would not be able to completely eradicate the pathogens from the body. Indeed, it has been observed that a fully functional immune system is also required to complete the eradication of the pathogens from the body [24]. This may also be achieved by combining antibiotics and phage therapy. Indeed, it has been noted that with some phages there is synergism between the phages and antibiotics and that phage-resistant variants sometimes become more susceptible to antibiotics [25,31].

## 5. Do Phages Threaten Beneficial Bacteria? 

As a consequence of the arms race between the bacteria and phages, the phage populations have been divided into subtypes that are specialized to certain subtypes of the bacteria. Therefore, the bacterial strains are classified as phage-sensitive and phage-resistant. This emergence of subtypes has proceeded such that within a single bacterial species there are only a few strains that are sensitive to a certain phage. Because of this, phages are typically regarded to possess a narrow host range. In practice, this means that in phage therapy each patient isolate has to be tested for suitable phages hopefully present in the laboratory phage collection, and that phage therapy is always precision medicine where the phages eliminate from the patients only those bacteria that are sensitive to the phage. The beneficial microbiome of the body will not be affected at all by the phages [32].

## 6. History of Phage Therapy

Bacteriophages have been known of for 100 years. The first observations of phage activity were reported by British researcher Ernest Hanbury Hankin in 1896 when he studied the influence of the water of the River Ganges on *Vibrio cholera* [33]. The water clearly killed the cholera bacteria, and this activity counted on the presence of cholera-specific phages in the water. During World War I, another British scientist, Fredrick Twort, and the French-Canadian scientist Felix d’Herelle first time detected clear bacteria-free lysis zones, plaques, within bacterial growth on solid culture media [34,35]. d’Herelle started to apply the phenomenon in practice to treat bacterial infections, and this is regarded as the beginning of phage therapy. Phage therapy was used all around the world with variable success until World War II, when penicillin and other antibiotics were invented. The use of antibiotics gradually ended the use of phages in the Western countries, but phage therapy continued actively in the Soviet Union. 

d’Herelle and Georgi Eliava founded a phage-institute in Tbilisi, Georgia in 1923. The institute started to produce phage preparates for diverse indications, and that activity still continues in Tbilisi [36]. Phage production was also started in several other cities in the Soviet Union, and has continued in present day Russia where the company MicroGen sells several phage products to be used for many indications [37]. Typically, such a phage product contains many different phages chosen for their ability to infect most bacterial pathogens isolated from patients. Thus, a product “Intesti-Bacteriophage” contains phages that infect and eliminate causative bacteria of intestinal infections such as bacteria of genuses *Shigella*, *Salmonella*, *Escherichia*, *Enterococcus*, *Proteus*, *Staphylococcus* and *Pseudomonas*. In Georgia and Russia, physicians can use phages in routine treatment of bacterial infections. This takes place specifically when the infection is not responsive to antibiotic treatment or if the patient is allergic to antibiotics.

While phage therapy is experiencing a rebirth in Western countries, the present legal issues on medicinal products places special requirements on phage therapy and phage products. This regulatory framework is not completely clear yet [38,39]. The problem is whether the phage product should be regarded as a medicinal product that should pass the same requirements as ordinary chemical drugs including clinical trials (phase I–III tests). If, on the other hand, phages are classified as active pharmaceutical ingredients (API), they could be included in the pharmacy as a treatment product able to be prescribed by a clinician [40].

A realistic view is that phage therapy is not a magic bullet that would be “The Cure of Everything”. It has its inherent limitations:

- Phage therapy as precision medicine implies that the causative agent of the infection is identified and cultured so that it can be tested for phage susceptibility.
- The interaction of a phage with the target pathogen may differ dramatically in vitro and in vivo in the body fluids, and that can only be tested using surrogate systems.
- To meet the demand, the phage therapy laboratory needs to possess a phage collection that covers all the most common pathogens. This could involve several thousand phages, the management of which requires skilled personnel and dedicated laboratory space. All the phages need to go through a thorough characterization before they can be approved for phage therapy [40]
- Production of safe phage products, even at a small personalized treatment scale, requires that approved quality control measures are in place. The sterility, stability, and the absence of endo- and exotoxins or other harmful compounds has to be ensured. In future, it is likely that phage products will be, at least partly, produced in dedicated GMP-facilities
- While natural phages are relatively easy to isolate against many pathogens, there are still many bacterial species for which phages are rare or even non-existent
- Work on phages against anaerobic bacteria or BSL class 3 pathogens presents additional challenges

## 7. Exploitation of Phages through Recombinant DNA Technology

In the context of phages, we also need to discuss (i) the (in)direct utilization of basic phage products as potential actors in therapeutic purposes, and (ii) the engineering of wild phages to better meet the need of therapeutic phages. 

With respect to the use of the phage products, endolysins have received most attention. Endolysins, with the help of holins, break down the bacterial cell wall and release the phage progeny to the environment at the end of the phage life cycle [41,42]. As endolysins specifically attack and degrade peptidoglycan, a lot of interest has been invested in their harnessing as antibacterial agents. While endolysins in general have relatively broad spectra, they nevertheless demonstrate distinct peptidoglycan specificities such that they may be restricted to a bacterial genus or family. In addition, due to the structure of the cell wall, Gram-positive bacteria are generally susceptible to externally administered endolysins, while that is not the case for Gram-negative bacteria where the outer membrane prevents the entry of the endolysin to the periplasmic space [42]. Due to their specificity for the pathogen, endolysins do not cause major disturbances among the normal microbiota.

Most phages encode products that take over the bacterial systems immediately after the injection of the phage genome into the bacterial cytoplasm. A detailed understanding of these effectors would likely open possibilities to develop drugs to their targets [43].

In many cases, suitable phages for patient isolates are not found, and then the question of the generation and production of recombinant or even synthetic phages becomes actual. A scenario for the year 2035 on this was recently presented [44]. It is conceivable that within the next 10–15 years enough data on phages and bacterial hosts will be accumulated to allow machine learning (artificial intelligence) to predict which phages would be suitable to treat pathogens isolated and sequenced from patients [45]. Regarding the synthetic phages, several promising approaches have been designed to that end [46], including, among others, an approach where the phage receptor binding protein specificity was expanded to a ‘phagebody’ library of 10^7^ variants [47].

Finally, in cases where suitable lytic phages are not available for a specific pathogen but temperate ones exist, DNA engineering can be utilized the delete the gene(s) involved in directing the phage to the lysogenic cycle. Thus, for example, the deletion of the repressor gene of two temperate *Mycobacterium* phages converted the phages into lytic ones that were subsequently used in the treatment of a lung transplantation patient who suffered from a *Mycobacterium abscessus* infection [48].

## 8. Experience on Phage Therapy in the World and in Finland

While phage therapy is in active use to treat bacterial infections under diverse indications in Russia and in Georgia strongly reflecting the historical situation, it has also been actively applied in Poland at the Hirzfeld Institute of Immunology and Experimental Therapy located in Wroclaw. There, starting in the 1950s, hundreds of patients have been treated successfully, with a cure rate close to 40% [49,50,51]. After Poland joined the European Union (EU) in 2004, phage therapy came under the legal framework of the EU, and at present treatments are only allowed for compassionate use. 

After 2000, increasing numbers of scientific reports have been published on experimental therapy trials that have used phages to cure infections caused by multidrug resistant (MDR) bacteria when the regular treatments have been unsuccessful. Typically, these treatments have not been conducted singly with phages, but phages have been combined with conventional treatments [25,48,52,53,54,55,56,57,58].

At the same time a few phase I and II clinical double-blind trials have been carried out with variable success [38,59]. Due to the narrow host range of phages it is difficult to recruit a suitable cohort of patients that would be infected by pathogens sensitive to the same phage. In addition, the administrators controlling the phage preparates have had little experience on the special requirements of phage therapy, and their initial demands have caused overwhelming difficulties [60]. Therefore, scientists have been forced to make compromises, and the goals of the trials have not been reached.

In my laboratory at the University of Helsinki, we have worked since 2013 with an aim to make phage therapy possible in Finland. The work has been carried out mainly via external funding but also with support from the Faculty of Medicine, University of Helsinki, and from the Helsinki University Hospital laboratory diagnostics. We are close to being ready to offer safe and efficient phage products to treat severe bacterial infections. The following subprojects have paved the way for this:

1. **Increasing the therapeutic phage collection.** It is recognized that the most dangerous bacterial pathogens for humans belong to the so called ESKAPEE-bacteria (*Enterococcus faecium*, *Staphylococcus aureus*, *Klebsiella pneumoniae*, *Acinetobacter baumannii*, *Pseudomonas aeruginosa*, *Enterobacter* spp., and *Escherichia coli*) that oftentimes show resistance towards most antibiotics, which makes the infections caused by them difficult to cure. Therefore, our primary aim is to isolate phages against these bacterial species. Phages are relatively easy to isolate; they are especially abundant and diverse in the sewage of cities and hospitals. Our phage collection at present comprises ca. 600 different phages. We have estimated that a collection of 1500–2000 different phages could cover 70–80% of the multidrug-resistant patient isolates. The isolated phages need to be thoroughly characterized before they can be included in the therapeutic phage collection [61]. This requires a lot of resources from the laboratory.
2. **Rapid phage typing method.** Phage therapy in the future will be precision medicine for which there are selected phages that are able to infect and kill the pathogen that has caused the infection. In practice, we screen our phage collection for suitable phages using a rapid method that also allows us to react to acute bacterial infections. To this end, we have set up a method based on liquid culture that can detect phage sensitivity in 3–4 h (unpublished results).
3. **Production of phage preparates.** One of the important goals has been to set up the processes to produce and purify phages for treatment. The purity requirements of the phage products are the same as those for other medical drugs, and there are border-values for possible contaminating impurities that should not be exceeded in the final preparate. We have optimized a purification process that requires no harmful chemicals and that can be scaled up upon request. For that we systematically tested several purification protocols in different combinations and the results were published in an article that raised wide interest [62]. In the protocol, using two purification methods, toxic bacterial remnants can be reduced to a safe level.

With our expertise and experience, we have prepared phage products for the treatments of ten patients suffering from different chronic infections caused by MDR pathogens. In all cases, the phage therapy has been safe, and for most patients the symptoms have been at least temporarily relieved (unpublished).

## 9. Future of Phage Therapy

While it looks like phage therapy is here to stay, there is still a lot to do. Clear instructions for actions and permissions for the treatments should be delivered by the medical authorities, and not just for experimental therapies. More practical experience on treatments should be collected, especially by clinicians that carry out the treatments. The curriculum of medical students does not include phage therapy yet, although it should be implemented there. Therefore, it is not imminent that phage therapy would become a common practice in the near future. Upon the accumulation of experience, knowledge of phage therapy will spread among physicians and among patients, and this will promote the position of phage therapy as a seriously considered alternative in the treatment of bacterial infections. Phages most likely will never replace antibiotics completely; however, they will be valuable in the treatment of infections caused by multidrug resistant bacteria. Antibiotics will still remain the main treatment for the majority of infections, especially the acute ones, for a long time.

In principle, phage therapy could in the future target any bacterial infection irrespective of whether the causative pathogen is sensitive or resistant to antibiotics as long as suitable phages are found for the pathogen. In practice, the use of precision treatments will require a seamless chain of events where the bacterial pathogen isolated from a patient sample, especially if it is MDR, will be transferred to a phage laboratory that screens the phage collection for suitable phages. In the best case, suitable phages can be ready-made products on the storage shelves. In such a case, a phage cocktail can be delivered to the clinician within a single working day. If the phage is not ready and immediately available, production and purification of the phage will take at least three to four days, to which a few days needs to be added for quality control. In the case that no phages are found in our collection, there is a possibility to get such from other phage laboratories around the world. The patient isolate can be shipped abroad in a couple of days and tested for suitable phages in the other collections. Finally, if this also fails, there is always a possibility to isolate phages from sewage samples. These last possibilities take from several days to weeks, so for the treatment of acute infections, the only possibility is that a suitable phage is already available. 
